# Functional Alignment of Regulatory Networks: A Study of Temperate Phages

**DOI:** 10.1371/journal.pcbi.0010074

**Published:** 2005-12-23

**Authors:** Ala Trusina, Kim Sneppen, Ian B Dodd, Keith E Shearwin, J. Barry Egan

**Affiliations:** 1 Department of Theoretical Physics, Umeå University, Umeå, Sweden; 2 Niels Bohr Institute, Copenhagen, Denmark; 3 Discipline of Biochemistry, School of Molecular and Biomedical Science, University of Adelaide, South Australia, Australia; Pennsylvania State University, United States of America

## Abstract

The relationship between the design and functionality of molecular networks is now a key issue in biology. Comparison of regulatory networks performing similar tasks can provide insights into how network architecture is constrained by the functions it directs. Here, we discuss methods of network comparison based on network architecture and signaling logic. Introducing local and global signaling scores for the difference between two networks, we quantify similarities between evolutionarily closely and distantly related bacteriophages. Despite the large evolutionary separation between phage λ and 186, their networks are found to be similar when difference is measured in terms of global signaling. We finally discuss how network alignment can be used to pinpoint protein similarities viewed from the network perspective.

## Synopsis

Networks of interacting genes and proteins orchestrate the complex functions of every living cell. Decoding the logic of these biochemical circuits is a central challenge facing biology today. Trusina et al. describe a mathematical method for aligning two regulatory networks based on their signaling properties, and apply it to a case study of three bacteriophages, simple biological “computers” whose genetics are exceptionally well characterized. The comparison reveals a surprising similarity between regulatory networks of the creatures, even when they have distant evolutionary relationships. The method introduced here should be applicable to other networks, and thus help to illuminate the computational substructures of living systems.

## Introduction

The functioning of living organisms is based on an intricate network of genes and proteins regulating each other. Various organisms differ due to not only differences in the constituting components (genes/proteins) but also the organization of these regulatory networks. It is, therefore, important to address similarities and differences in not only protein sequences but also the interaction patterns of the proteins. Thus, large-scale analysis of protein–protein and protein–DNA interactions have provided insight into the local design features of subcellular signaling [1–3]; network alignment based on sequence similarities permits alignment of related motifs [4,5].

Here we suggest comparison of networks through an alignment method that is based solely on network architecture and signaling logic, which thus does not rely on sequence similarity of the involved proteins.

As a case study, we considered the regulatory networks of two well-characterized temperate bacteriophages of *E. coli,* λ and 186 (Figure 1). These two phages represent two distinct classes of temperate bacteriophages: the lambdoid phages—which include λ, P22, 434, HK97, and HK022, and the P2 group—which includes P2, 186, HP1, K139, and PSP3. λ and 186 are not detectably related in sequence and have different genome organizations. Using tBLASTx [6] to compare all of the reading frames, there are only two clearly homologous protein pairs: the λ endolysin R/186 (E-score = 10^−34^) and a pair of early lytic proteins of unknown function (E-score = 2 × 10^−4^). No significant similarity was detectable at the nucleotide level (using BLASTn, [6]). On the genome level, the arrangement of genes, promoters, and operators is very different [7–10]. As a control of methodology, we also considered the P22 phage, which, as a member of the lambdoid family, allows us to compare topologies of evolutionarily related networks.

**Figure 1:**
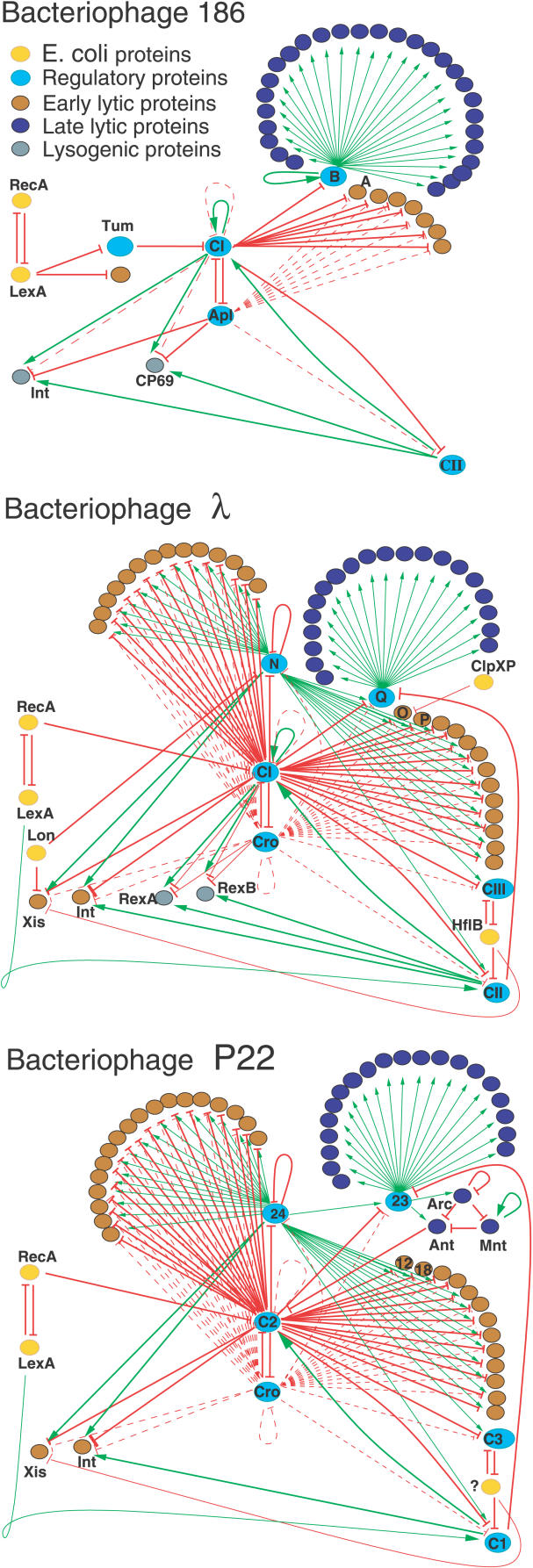
The Genetic Regulatory Networks for Phage 186, Phage λ, and Phage P22, All of Which Are Temperate and Infect *E. coli* The proteins are colored according to their functions and expression mode in the lysis–lysogeny life cycle of the phages. We summarize the influence of one protein on another by either a green arrow (positive, e.g., transcriptional activation) or a red arrow (negative, e.g., repression). The dashed lines show relatively weak regulations.

As a temperate phage, 186 and λ each can be in two states: a lytic state where many proteins are active in the replication of the phage DNA and the construction and release of virus particles; and a lysogenic state where the phage genome is integrated into the bacterial chromosome and only a few proteins are active. For both phages, three core proteins (CI, Cro, and CII in λ, and CI, Apl, and CII in 186) do the main computations, with the switch into lysogeny being coordinated by CII and the reverse switch into the lytic mode initiated by activation of the host SOS response recombination (RecA) protein. The gene-regulatory networks of all temperate phages have evolved to provide lysogenic and lytic states, and, moreover, to switch from one state to another when particular signals have been received from bacterial proteins, and thus effectively perform the same function.

Given that 186 and λ are both temperate, i.e., performing a similar function, but evolutionarily separated, we asked whether we could detect structural similarities, and at what scale these similarities were detectable.

## Results

Visual comparison of the 186 and λ networks (see Figure 1) suggests both strong similarities but also major differences. One way to quantify the similarity of two networks is by edit distance [11]. Assume that we know which nodes (here, proteins) in networks A and B should be paired. For networks of the same size, we define edit distance as the number of insertions or removals of edges (regulatory connections) one has to perform on network A to obtain B. This is quantified as


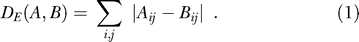


The elements *A_ij_* and *B_ij_* specify whether the direct regulation of *i* on protein *j* is positive, negative, or absent, and are constructed such that each element can keep both positive and negative links (for details, see equation 2 below).

In case we do not know which nodes in networks A and B should be paired, we find the optimal identification by minimizing *D_E_* as described in the Materials and Methods section. This yields the minimal distance between the networks, as well as an optimal alignment of the individual nodes. We call this distance the edit difference.

The minimal edit difference between *related* phages is small, *D_E_* (λ, P22) = 18, compared with the larger scores for evolutionarily separated phages (Table 1). *D_E_* = 18 means that the λ network of 62 proteins and 144 connections can be constructed by making 18 edits of the connections in a 62-protein subset of the 67-protein P22 network (adding or removing a link is a single edit; changing the sign of a connection is two edits). To get an idea of the significance of the obtained *D_E_* values, we compared them with optimal alignments of 500 randomized versions of the two networks. The randomization procedure was designed to conserve the local properties of the networks to try to keep their general biological features. First, the core-hub topology common in biological networks [3] was maintained by conserving for each protein the number of its regulators (inputs) and the number of proteins regulated by it (outputs). Second, the number of each sign (positive and negative) of the input and output connections was kept for each node.

**Table 1:**
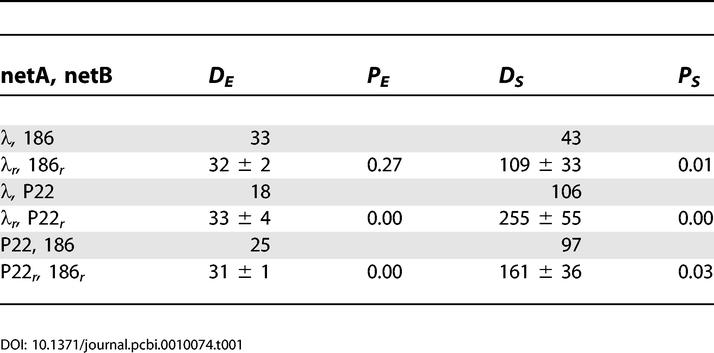
The Overall Difference Measures, *D_E_, D_S_,* between the Networks, with Respective P-Scores as Defined in Text

The constraint of preserving the local properties does not fix the network completely: while keeping the number of positively or negatively regulated proteins, one can still change which of them are being regulated. The structure of the resulting random networks is rather different, as seen in the examples shown in Figure 2.

**Figure 2:**
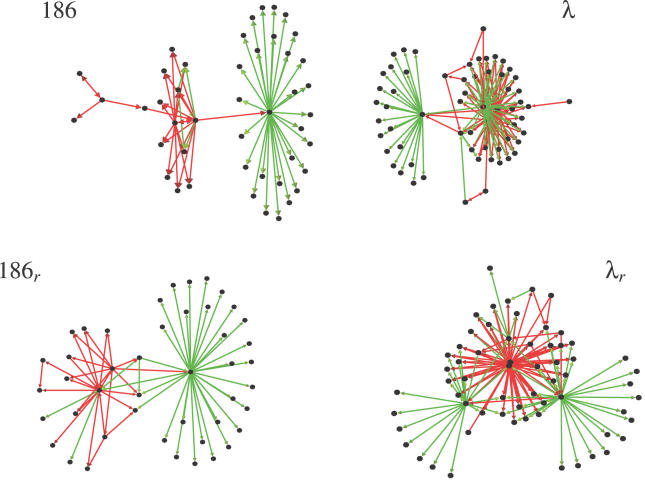
Illustration of the Differences between the Real 186 and λ Networks (Top) and an Example of Their Randomized Counterparts (Bottom) These examples of randomized networks show that it possible to preserve local properties, yet obtain different network structures.

Overall, we found that *D_E_* scores between any pair of randomized networks are similar. When comparing scores between real networks with those of their random counterparts in Table 1, one sees no clear trend. In particular, the differences between these randomized versions for λ*_r_* and 186*_r_* were indistinguishable from those of the real networks: *D_E_* (186*_r_,* λ*_r_*) = 32 ± 2.

We reasoned that the functional similarity of networks might be better reflected in a less local measure of functionality. We therefore introduced a signaling difference, *D_S_,* which aims to capture both direct (as in *D_E_*) and indirect regulation through a sequence of intermediate proteins. For each pair of proteins *(i, j),* we considered whether *i* sends a signal to *j,* and if so whether the signal along the shortest path is positive or negative. In this spirit, we define the sign of a signal as the product of the signs of all links on the shortest path from *i* to *j*. An example where this procedure nicely reflects the functionality in terms of its Boolean logic [12] is found in the pathway from RecA to CI in the two phages. In λ, active RecA directly catalyzes self-cleavage of CI [13]; whereas in 186, RecA acts through the degradation of a repressor protein (LexA), which in turn represses the protein Tum [9], which in the absence of repression binds CI and prevents it from performing its function. Thus, the simple −1 signal in λ is in 186 replaced by a signaling consisting of (−1) × (−1) × (−1) = −1. In other words, repressing a repressor is effectively an activation.

Because the regulation of one protein by another may be positive through one series of links and negative through another, two matrices were used for each network, one for positive signals (*A^S+^* and *B^S+^*) and one for negative signals (*A^S−^* and *B^S−^*). If the effect of protein *i* on protein *j* is only positive, then one is placed into 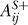 and zero into 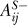 . If the effect is only negative, then zero is placed into 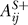 and one into 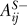 . If there are positive and negative signals along paths of equal length (e.g., from RecA to λ CII via LexA or CI), then one is placed into both matrices. Observe that when positive and negative signals come to the same node, they are not canceling each other. This is intentional, as often signals will arrive at different times or at different conditions. (An example of this is the two paths from RecA to CII over CI and LexA, respectively, of which only the RecA-LexA-CII path is activated during lysis.)

The signaling difference between two networks A and B is then defined as


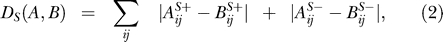


which takes into account differences in both positive and negative signaling along the shortest paths between any pair of nodes. Like *D_E_,* the minimum difference *D_S_* is calculated by optimizing which proteins in 186 should be identified with which proteins in λ, and, in addition, which λ proteins should be excluded. Excluding a protein means that the signaling to and from that protein is not counted in *D_S_,* whereas signaling across the excluded protein is included.

Optimizing protein alignment based on signaling, we found that *D_S_* (186, λ) = 43. Again, the significance of this difference was determined by repeatedly performing randomization of the networks as described above, creating the *A^S+^* and *A^S−^* matrices and obtaining the minimal *D_S_*. The differences between random networks, *D_S_* (186*_r_,* λ*_r_*) = 109 ± 33, is much larger than between the real networks. This is further quantified by a P-score, P (*D_S_* > *D_S_* (random)) = 0.01, defined as the probability that two randomized networks will have a smaller difference than that between the real networks.

Thus, all three networks are similar in their signaling pattern. To confirm that this signaling similarity is not generally conserved among biological networks, we compared the phage networks with other networks that perform different functions (e.g., the *Saccharomyces cerevisiae* cell-cycle network [14], and the *Bacillus subtilis* competence networks [15]). We found that *D_S_* is much larger and the P-scores are close to one in these alignments, indicating that the low signaling difference between the phage networks is a special property of these functionally similar networks.

We also considered other variants of the difference measures, in particular including all non-repetitive paths between pairs of proteins, with all paths weighted equally. In that case, we also found that *D_S − all_* (λ, 186) = 390 between real networks is smaller than *D_S − all_* (λ*_r_,* 186*_r_*) = 583 ± 122 between the randomized counterparts. Also, using the shortest paths, we investigated differences between networks where weak links (see the dashed ones in Figure 1) are weighted less (by a factor of 0.5 or removed altogether). *D_S_* scores between networks got smaller, but overall significance remained similar.

## Discussion

The pathway-related *D_S_* score allowed us to identify significant similarity between two distantly related biological networks (see Table 1). In contrast, the edit difference measure, which looks only at the local wiring structure, is sometimes blind to this more global “homology.” Thus, although edit difference partially captures network similarities through a patchwork of local matchings, it is less sensitive to pathway disruptions.

It is not clear whether the functional similarity between the λ and 186 networks detected by the *D_S_* measure is a result of convergent evolution or is a remnant of a shared ancestral network. In either scenario, it is clear that the two network structures must be strongly constrained by functional requirements, given the evolutionary separation of the two phages. A potential bias should be noted here: knowledge of the three phage networks is not complete, even for λ, and it is thus possible that some of the observed similarity in the networks is due to knowledge of connections in one phage network having influenced the discovery of connections in the others.

The *D_S_* alignment allows us to address the role of various proteins in pathway disruptions. Figure 3 lines up the λ and 186 proteins on the basis of pre-existing knowledge of their function or mode of expression and indicates the optimal *D_S_* alignment and the contribution of each pair to the signaling difference. The two alignments show good matches for late lytic genes as well as for the regulators CI, CII, and B from 186 aligned with CI, CII, and Q in λ. Thus, in general, functions of proteins in one network teach us about protein properties in the other network. The lack of a good match between Apl (in 186) and Cro (in λ) is due to the weak links from Cro and reflects a different functional role of Cro and Apl in the late lytic development of phages. Insisting on alignment of Cro with Apl results in *D_S_* = 219, thus emphasizing the particular role of Cro as a repressor of late lysis in λ.

**Figure 3:**
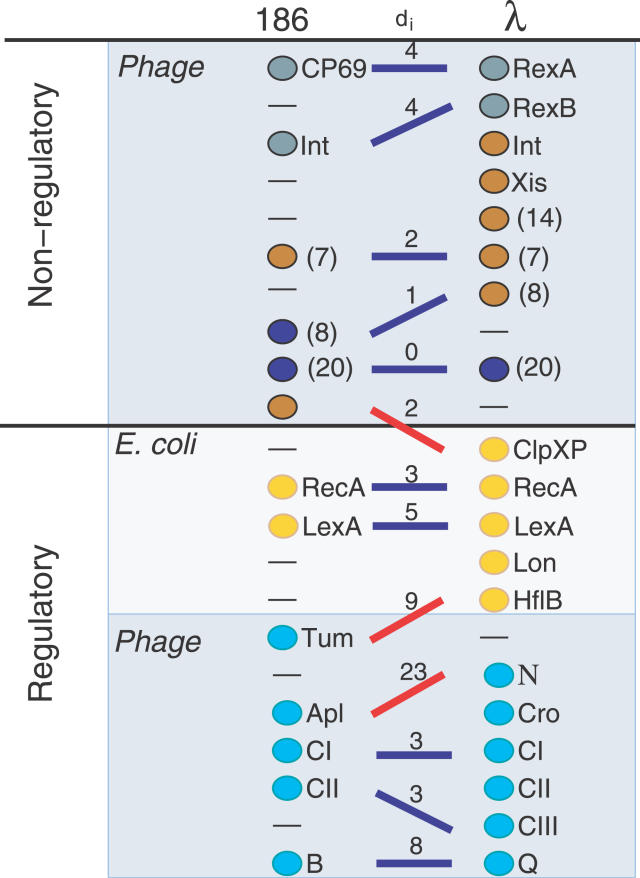
Alignment of Two Phage Networks Placement of proteins is based on our knowledge [7–9,13,22], and the lines connecting them are associated with the minimal *D_S_* alignment. Proteins that perform similar functions or are regulated similarly are placed on the same level; thus, horizontal lines mark ideal matching. Blue lines correspond to meaningful alignments, and red lines are the misalignments. The numbers above the lines, *d_i_,* reflect the differences in signaling between the aligned proteins and are the contributions to the minimal difference 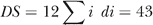 . The numbers in the parentheses indicate multiple equivalent proteins, making the sum of all shown signaling differences equal to 2 × 43. The key regulators RecA, LexA, and CI are identified correctly, whereas the misidentification of CII with CIII is reasonable since both favor entry into lysogeny through the same pathway. The major discrepancy is associated with the different roles of Cro and Apl during lysis (the weak links from Cro to Q and N in λ).

Comparison of molecular networks is becoming an important element of modern systems biology, both with regard to predicting eventual missing links [16], and to increasing our understanding of functionality of information processing in the networks. The alignment methods presented here address the similarities on a local, respectively larger scale, associated with signaling across networks.

In this regard, we found that evolutionary relationships (λ − P22) imply similar local regulation, with a low *D_E_* score. For all temperate phages, evolved to do similar “computation,” their regulatory networks are found to be similar when viewed from a more global perspective where both direct and indirect signals are included (low *D_S_* score compared to random expectation). Thus the mechanistic and structural differences on the scale of genome and promoter organization disappear when considering the large scale of the protein regulatory networks. Going beyond immediate regulations allows us to capture functional similarity in the most robust way.

## Materials and Methods

The present paper is based on the data on three bacteriophages: λ, P22, and 186. The regulatory networks were compiled from these database entries and various literature sources: for λ ([7,13,17,18,] and references therein), for 186 ([8–10] and references therein), and for P22 ([19] and references therein).

In the Results section, we define two differences scores, *D_E_* and *D_S_,* between a pair of networks *A* and *B*. Provided that we know which proteins in *A* should be identified with which in *B,* the scores are calculated as in equation 1 and equation 2. In case we do not know which nodes in networks *A* and *B* should be paired, we need to find the optimal identification of nodes between them. To do so, we define an alignment procedure through the Metropolis Algorithm [20], designed to reach the minimal distance *D* between the networks: given two nodes and their corresponding partners in the other network, the elementary step is to switch partners and re-evaluate the distance. Iterating this procedure and using simulated annealing [21], the difference score between the two networks converges to a global minimum.

If the two networks are of different sizes, we count only the contribution from a number of nodes given by the smaller of the two networks. In the larger network, these nodes are selected to minimize the distance using the above algorithm.

We would like to note that the above method is not intended to reflect any evolutionary process, but is used to find the optimal mapping of pairs of proteins that look similar from the network perspective. The method is limited by the network size, and in practice it works for networks of fewer than 200 nodes.

The realization of the alignment algorithm in the form of the Java applet (Sun Microsystems, Santa Clara, California, United States) is available at http://www.cmol.nbi.dk/models/compar/compar.html.

## Supporting Information

### Accession Numbers

The GenBank (http://www.ncbi.nlm.nih.gov) accession numbers for the genes and gene products discussed in this paper are: P22 (NC002371) and λ (J02459).

The Swiss-Prot (http://ca.expasy.org/sprot) accession numbers for genes and gene products discussed in this paper are: 186 (PO80309), Apl (P21681), CI (P03034 and P08707), CII (P03042 and P21678), CIII (P03044), Cro (P03040), LexA (P03033), R (P03706 and PO80309), RecA (P03017), and Tum (P41063).
